# Advance in the Diagnostics and Management of Musculoskeletal Diseases

**DOI:** 10.3390/diagnostics12071588

**Published:** 2022-06-29

**Authors:** Alessandro de Sire, Elisabetta Ferraro, Massimiliano Leigheb

**Affiliations:** 1 Department of Medical and Surgical Sciences, University of Catanzaro “Magna Graecia”, 88100 Catanzaro, Italy; 2 Cell and Developmental Biology Unit, Department of Biology, University of Pisa, 56127 Pisa, Italy; elisabetta.ferraro@unipi.it; 3 Orthopaedics and Traumatology Unit, “Maggiore della Carità” Hospital, Department of Health Sciences, University of Piemonte Orientale, 28100 Novara, Italy; massimiliano.leigheb@uniupo.it

## 1. Introduction

Musculoskeletal disorders are a broad spectrum of diseases, affecting muscles, bones, ligaments, and tendons worldwide. The interaction between professionals in a multi- and transdisciplinary field is very important as these pathologies must be addressed by more professionals than basic scientists, professionals such as orthopedists, traumatologists, physiatrists, physiotherapists, rheumatologists, geriatricians, radiologists, biologists, biotechnologists, and bioengineers [1,2,3].

In recent years, the number of prevalent cases of musculoskeletal disorders in need of rehabilitation increased dramatically; they are now a common cause of pain and functional disability [1].

An early diagnosis is often crucial for an optimal management of inflammatory musculoskeletal diseases (e.g., osteoarthritis, low back pain, temporomandibular disorders), and in most cases both clinical, biological, and radiological evaluations are all needed. To date, imaging and biomarkers (i.e., cytokines) have received growing attention by scientists [4,5], with a significant progress in novel therapeutic approaches (e.g., oxygen-ozone therapy) to treat pathological disorders characterized by chronic inflammatory processes and immune hyper activation [6,7,8].

Therefore, this Special Issue is aimed at summarizing the state of the most interesting diseases of the musculoskeletal system, and to describe the most recent advancements in diagnostic tools and in the management of these conditions.

## 2. Diagnostics for Muscular Diseases

Sarcopenia is defined as a progressive and generalized skeletal muscle disorder, characterized by a low muscle mass and function [9]. While muscle mass is commonly evaluated by Dual-energy X-ray absorptiometry (DXA), Bioelectrical Impedance Analysis (BIA) and lumbar muscle cross-sectional area by Computed Tomography (CT) or Magnetic Resonance Imaging (MRI), Park et al. [10] tested the feasibility of 18F-fluorocholine (18F-FCH) positron emission tomography/computed tomography (PET/CT) in muscle mass evaluation, tracking choline level changes in rat skeletal muscles. In fact, choline is crucial for muscle contraction, as precursor to acetylcholine, the major neurotransmitter in alpha motor neurons. The authors found that 18F-FCH uptake was lower in the atrophy-induced cells compared to untreated ones, and in-vivo PET uptake also revealed a similar trend with average standardized uptake values (SUV) of 0.26 ± 0.06 vs. 0.37 ± 0.07. Similarly, intriguing results were reported by Leigheb et al. [11], who assessed the reliability of ultrasound thickness and quality parameters, as echogenicity and stiffness of tibialis anterior (TA) muscle, in sarcopenia diagnosis in a cohort of 33 bedridden patients due to orthopedic surgery. They found that ROC curves predicted reduced muscle strength and mass for TA thickness (0.89 and 0.97 respectively) and for TA stiffness (0.73 and 0.85 respectively); thus, ultrasound might be considered as a reliable measurement tool in sarcopenia diagnosis. Other than muscle mass, muscle strength should also be evaluated for sarcopenia diagnosis, albeit guidelines only suggest sit-to-stand tests and hand grip strength test, with the latter showing wide variability among subjects [9]. In this context, Trajković et al. [12] investigated the interrater and intra-rater reliability of a belt stabilized handheld dynamometer measuring isometric strength of 23 young adults in knee extension, knee flection, hip adduction, hip abduction, shoulder internal rotation, and shoulder abduction. The results supported the good to high inter- and intra-rater reliability with an intraclass correlation coefficient range of 0.63–0.91.

For muscle disease diagnosis, muscle biopsy with histological and morphometric analysis might be necessary to confirm the clinical suspect. Non-automatic methods are time-consuming and prone to error, while Laghi et al. [13] proposed an automatized computational approach utilizing a specific software, which was tested on a mouse model of the genetic myopathy Duchenne Muscular Dystrophy. By this approach, measuring and quantifying the minimum Feret diameter, centrally nucleated fibers, the number of macrophages, and the extracellular matrix in skeletal myofibers is quick and reliable compared to manual approaches, thus displaying a higher feasibility and reproducibility.

## 3. Diagnostics in Ligament and Tendon Injuries

With regard to tendon injuries, among the broad spectrum of inflammatory and degenerative processes, the most common acute condition is the rupture, particularly in athletes [14]. In particular, the most common tendon involved is the Achilles, with an incidence of nearly 40/100,000 in athletes, and rotator cuff [14].

Dramatic events in athletes are also sprains and tears of ligaments that could cause disability and pain, requiring significant time and resources from diagnosis to rehabilitation, for the return-to-play. Indeed, the diagnosis might require ultrasound or MRI other than clinical exams, for both tendon and ligament injuries [15]. Ligament injuries involved commonly comprehend the ankle and knee region with the latter associated with capsular or meniscus injury in most cases; from an epidemiological point of view, Anterior Cruciate Ligament (ACL) injuries could reach an incidence of 29–38/100,000 in athletes [15]

ACL injuries are among the most disabling sports-related issues, occurring with sudden pivoting or cutting movements [16]. Recently, technological advancements have also drastically influenced rehabilitation approaches, providing novel techniques. A study performed by de Sire et al. [17] introduced the role of visual input on of the rectus femoris, vastus medialis, biceps femoris, and medial hamstrings muscles pre-activation evaluated through surface electromyography (sEMG) integrated in the rehabilitation training to reduce ACL injury risk in tennis players. The results showed an increased muscle activation with higher medial hamstring/rectus femoris ratio when blindfolded or landing on unsafe terrain. This confirms the crucial role of visive input and neuro-activation protecting effect, which can be trained with sEMG during the athletic preparation.

Furthermore, in ACL injuries, other than commonly performed clinical tests and static MRI, a recent study by Csapo et al. [18] investigated the possibility to perform a magnetic resonance during the Lachman-like knee stress test to measure elongation, on a model human cadaveric leg. The elongation raged between 0.7 and 1.7 mm and agreed with in situ data, with a root mean square error of 0.19–0.36 mm/s. This dynamic method might be promising in evaluating potential strain and ACL loading capacity.

Although MRI is commonly utilized in ligament injuries, in anterior talofibular ligament (ATFL) injuries, diagnosis is based mainly on pain and chronic instability. Barini et al. [19] performed a systematic review and metanalysis aimed at investigating the accuracy of MRI in this condition. Authors selected seven studies meeting the inclusion criteria and found that the pooled sensitivities and specificity in diagnosing acute ATFL injury were quite high—respectively, 1.0 (95% CI: 0.58–1) and 0.9 (95% CI: 0.79–0.96)—suggesting that with ATFL acute injury a routine MRI could be useful, although it is not currently performed in clinical practice.

## 4. Diagnostics in Bone Diseases

Osteoporosis is a systemic bone disease characterized by low bone mass and microarchitectural deterioration of bone tissue, and increased risk of fractures [20]. Osteoporosis commonly manifests in post-menopausal age, but it is also associated with rare genetic diseases [18]. Indeed, low bone mass should also be investigated in connective tissue syndromes, as described in a recent case report by Cortés-Martín et al. [21], who diagnosed a 54-year-old woman with craniofacial dysmorphia, short stature, premature loss of teeth, developmental skeletal disorders, fibrocystic mastopathy, bilateral hearing loss, and an intermittent mild neutropenia, and who was diagnosed with Hajdu-Cheney syndrome, a rare disease with a global prevalence of less than one person out of one million worldwide. Hajdu-Cheney syndrome is caused by a mutation in the NOTCH2 gene, and follows an autosomal dominant inheritance, although only a hundred cases are known and described in the literature.

To date, biochemical markers are particularly useful in the diagnosis and management of osteoporosis, as they reflect bone resorption and apposition. While fat tissue seems to have a protective effect on bone loss, evidence exists that the risk of non-vertebral fractures can be increased in postmenopausal women. In this scenario, Marzullo et al. [22] investigated the potential role of body composition in 28 severely obese premenopausal women, compared with obese post-menopausal controls, evaluating DXA, bone turnover markers, sclerostin serum concentration, glucose metabolism, and hormones, harboring that sclerostin was the strongest predictor of lumbar spine bone density, and lean mass was the stronger predictor of total body bone density, meaning that fat mass might be effectively beneficial on bone. While appendicular and body fat mass might be a protective factor, bone marrow adiposity is recognized as substitute of bone mineral content in case of low BMD, particularly in patients with chronic kidney diseases. Therefore, Chen et al. [23] investigated the role of adiponectin as fat-bone critical mediator in patients subjected to maintenance hemodialysis. Authors found that adiponectin, phosphate, and intact parathyroid hormone were significantly associated with higher risks of fracture. Interestingly, adiponectin serum levels showed a cutoff point concentration of 18.15 ug/mL with a ROC curve prediction of 0.66 (95% CI = 0.49 to 0.84).

Vertebral and hip fractures are the most characterized osteoporosis fracture in literature, as the latter also frequently occurs in older people without osteoporosis and often associated with devastating effects on functioning and independence [24]. Indeed, hip arthroplasty is the most common intervention for joint replacement, but there is no consensus in the literature on the real influence of this procedure on balance. A recent systematic review performed by Di Laura Frattura et al. [25], on 27 papers and 391 patients, suggested that balance impairment occurs immediately after surgery, and at 4–12 months after patients reach better balance levels; these findings highlight the importance of balance assessment.

Regarding balance and fractures, ankle fracture primarily causes gait cycle alteration and unbalance. In this context, gait assessment might be useful in this condition, as described by Mirando et al. [26] in a recent systematic review, including 12 studies, aiming to objectively characterize through gait analysis the alterations in walking. The authors found a reduction in step length, swing time, single support time, stride length, cadence, speed, and an earlier foot-off time in the affected side. Additionally, trunk movement symmetry was significantly reduced. These findings encourage a broad gait analysis utilization in performance evaluation.

Although fractures are primarily caused by high trauma impact, or by low trauma in pathological condition, some therapeutic maneuvers might as well cause cervical vertebrae fracture, if performed carelessly, as the cervical vertebral manipulation [25]. Indeed, Bernetti et al. [27] performed a systematic review concerning the effects and the adverse events of cervical spine manipulation in the physical and rehabilitation medicine field with a forensic medicine perspective. The authors recommended that this therapeutic procedure should be performed only by professionals, after an accurate diagnostic procedure.

Finally, as an underestimated consequence of trauma or orthopedic intervention, Complex Regional Pain Syndrome type I (CRPS I) might occur, with typical findings consisting in severe pain, functional limitation, and vasomotor alteration [28]. In some cases, however, this condition might also be related to non-orthopedic procedures, as depicted by Moretti et al. [29] in a recent case report, describing a case of CRPS I spontaneously following percutaneous transluminal coronary angioplasty, in a 44-year-old man approximately 5 months after the event. Bone scan might be crucial in diagnosing controversial cases, along with a comprehensive clinical and instrumental evaluation.

## 5. Conclusions

Taken together, this Special Issue highlights the importance of clinical and instrumental investigations for the diagnosis of diseases of muscles, ligaments, and bones diseases. Diagnostic procedures should not be limited to traditional radiology, but they should also include electromyography, gait analysis, dynamic MRI and ultrasound scans, contrast enhancement utilization, novel laboratory biomarkers, and computed software. Therefore, physicians should be aware of the presence of novel diagnostic approaches that are changing the management of patients affected by musculoskeletal disorders.

## Data Availability

Not applicable.
